# Multifractal Analysis Reveals Decreased Non-linearity and Stronger Anticorrelations in Heart Period Fluctuations of Fibromyalgia Patients

**DOI:** 10.3389/fphys.2018.01118

**Published:** 2018-08-17

**Authors:** Cesar F. Reyes-Manzano, Claudia Lerma, Juan C. Echeverría, Manuel Martínez-Lavin, Laura A. Martínez-Martínez, Oscar Infante, Lev Guzmán-Vargas

**Affiliations:** ^1^Unidad Profesional Interdisciplinaria en Ingeniería y Tecnologías Avanzadas, Instituto Politécnico Nacional, Ciudad de Mexico, Mexico; ^2^Departamento de Instrumentación Electromecánica, Instituto Nacional de Cardiología Ignacio Chávez, Ciudad de Mexico, Mexico; ^3^Departamento de Ingeniería Eléctrica, Universidad Autónoma Metropolitana Unidad Iztapalapa, Ciudad de Mexico, Mexico; ^4^Departamento de Reumatología, Instituto Nacional de Cardiología Ignacio Chávez, Ciudad de Mexico, Mexico

**Keywords:** heart rate variability, scaling, magnitude and sign analysis, complexity theory, dysautonomia, multifractaility, fibromyalgia

## Abstract

**Objective:** To characterize the multifractal behavior of the beat to beat heart-period or RR fluctuations in fibromyalgia patients (FM) in comparison with healthy-matched subjects.

**Methods:** Multifractral detrended fluctuation analysis (MDFA) was used to study multifractality in heartbeat times-series from 30 female healthy subjects and 30 female patients with fibromyalgia during day and night periods.The multifractal changes as derived from the magnitude and sign analysis of these RR fluctuations were also assessed.

**Results:** The RR fluctuations dynamics of healthy subjects showed a broad multifractal spectrum. By contrast, a noticeable decrease in multifractality and non-linearity was observed for patients with fibromyalgia. In addition, the spectra corresponding to FM subjects were located on the average to the right of the spectra of healthy individuals, indicating that the local scaling exponents reflect a smoother behavior compared to healthy dynamics. Moreover, the multifractal analysis as applied to the magnitude and sign heartbeat series confirmed that, in addition to a decreased nonlinearity, fibromyalgia patients presented stronger anticorrelation in directionality, which did not remain invariant for small or rather larger fluctuations as it occurred in healthy subjects.

**Conclusion:** When compared to healthy controls, fibromyalgia patients display decreased nonlinearity and stronger anticorrelations in heart period fluctuations. These findings reinforce the hypothesis of the potential role of the dysfunctional autonomic nervous system in the pathogenesis of fibromyalgia.

## 1. Introduction

Diverse methods derived from non-linear dynamics and statistical physics have been used to characterize the spatiotemporal organization displayed by complex signals from different living systems (Ashkenazy et al., 2002; Hu et al., 2004; Ivanov et al., 2001, 2009; Bassingthwaighte et al., 2013). Frequently, many of these signals exhibit power law scaling when analyzed by techniques which are capable to detect a single scaling exponent; but for other signals, a better description is given in terms of a set of local exponents as it is the case of multifractals (Goldberger et al., 2002; Bunde et al., 2012). The multifractal formalism was introduced in the context of turbulence studies (Kolmogorov, 1941; Frisch, 1995) and velocity fluctuations (Frisch and Parisi, 1985). Since Mandelbrot (1974) introduced the concept of multifractality in the context of geometric objects; many different systems have been described using a broad multifractal spectrum, indicating that a wide range of local exponent values are needed to characterize the irregularity in the original signal (Mandelbrot, 1977; Ivanov et al., 1999a, 2001; Feder, 2013). The traditional procedure to perform a multifractal analysis is derived from the construction of a standard partition function, and only applies to normalized stationary signals (Wang et al., 2014). At the beginning of the 90s, the wavelet transform modulus maxima (WTMM) method was introduced to determine the multifractal spectrum based on wavelet transform over different scales (Daubechies, 1992; Bacry et al., 1993; Muzy et al., 1993, 1994; Arneodo et al., 1995a,b; Ivanov et al., 1996).

Later, in 2002 Kantelhardt et al. (2002) introduced the multifractral detrended fluctuation analysis (MDFA), as an extension of the monofractal detrended fluctuation analysis (DFA) method (Peng et al., 1994). One of the advantages of the MDFA is that it provides a stable spectrum of a range of multifractal signals, with a reliable estimation of the set of local Hurst exponents. Besides, the MDFA has been tested to extract a reliable multifractal spectrum when it is applied within time scales corresponding to low frequencies (Galaska et al., 2008).

On the other hand, the RR fluctuations or heart rate variability has been the object of study by means of nonlinear methods during past decades (Nunes Amaral et al., 2001; Ivanov et al., 2001, 2009; Peña et al., 2009; Hernández-Pérez et al., 2011). One of the most important features extracted by means of these methods is the presence of power-law fractal organization under healthy circumstances, while a degradation of the fractal scaling is frequently observed for pathologic conditions (Guzmán-Vargas et al., 2003). A more detailed assessment of the complex RR fluctuations have revealed that healthy interbeat dynamics is well described by a broad mutifractal spectrum, and a reduction in the multifractality was detected for patients with congestive heart failure (Ivanov et al., 1996; Guzmán-Vargas and Angulo-Brown, 2003; Guzmán-Vargas et al., 2005; Bojorges-Valdez et al., 2007; Galaska et al., 2008).

Given the need to elucidate the etiology of fibromyalgia (FM), identifying alterations in its RR fluctuations complexity has gained particular interest. In this pathology, chronic pain and other symptoms affect multiple systems extensively (Martinez-Lavin et al., 2008). The pathophysiology of FM remains uncertain, and no specific mechanisms can be pinpointed to explain the dynamical changes observed in the heart rate variability of these patients. However, there is strong evidence about the involvement of the autonomic nervous system both, in the etiology and the multifaceted alterations of this disease. The main affliction of the syndrome (chronic pain) seems to be maintained by the chronic sympathetic hyperactivity that is associated with altered connections between the sensory neurons and the sympathetic nervous system (Martinez-Lavin, 2004). In addition, the fact of having defective catecholamine clearing enzymes appears to increase the susceptibility to pain (Diatchenko et al., 2004). We have previously hypothesized that the understanding of FM requires an approach in which the autonomic nervous system is considered as a complex adaptive system, which in itself constitutes the main element of the stress response system (Martinez-Lavin et al., 2008). From this point of view, we would expect that the alterations in this complex adaptive system become manifested in the dynamical properties of the physiological variables that such system regulates (such as the beat to beat heart-period or RR fluctuations). The first evidence of an altered complexity of RR fluctuations in FM was the observation of a larger monofractal short-term scaling exponent in FM patients compared to healthy subjects (Lerma et al., 2016). Yet, it is not known if the multifractal spectrum of these patients also shows a reduction or other alterations. Here, we studied RR time series of patients with FM during day and night periods. Our aim was to characterize the multifractal behavior of the RR fluctuations in FM patients and in carefully selected healthy-matched subjects, using the MDFA method. In addition, the multifractal changes as derived from the magnitude and sign analysis of these RR fluctuations were also assessed.

## 2. Methods and data

### 2.1. Multifractal detrended fluctuation analysis (MFDFA)

The multifractal detrended fluctuation analysis was introduced by Kantelhardt et al., which is a robust method to detect the scaling properties of the fluctuactions related with multifractilty of a given signal. We briefly explain the main steps of the multifractal detrended fluctuation analysis (Kantelhardt et al., 2002):

- Step 1: Given the time series *x*_*k*_ of length *N*. First, we determine the profile
  (1) $$\begin{array}{l} Y\left(i\right)\equiv \overset{i}{\underset{k=1}{\sum }}\left[x_{k}-\langle x\rangle \right],\;i=1,\ldots ,N, \end{array}$$
  where 〈*x*〉 represents the mean value.
- Step 2: Next, the profile *Y*(*i*) is divided into *N*_*s*_ ≡ *int*(*N*/*s*) segments of size *s*. To make more robust the statistics, the same procedure is applied but starting from the end of the time series, and in this way, 2*N*_*s*_ segments are considered for the calculations.
- Step 3: A least-square fit is applied to the 2*N*_*s*_ segments of the integrated data. Then the variance is calculated,
  (2) $$\begin{array}{l} F^{2}\left(v,s\right)\equiv \frac{1}{s}\overset{s}{\underset{i=1}{\sum }}{\left\{Y\left[\left(v-1\right)s+i\right]-y_{v}\left(i\right)\right\}}^{2}, \end{array}$$
  for, *v* = 1, …, *N*_*s*_, where *y*_*v*_ represents the fitting polynomial, which can be linear, quadratic, or a higher order polynomial.
- Step 4: Now the *q*th-values are considered in the fluctuation function
  (3) $$F_{q}(s)\equiv \;{\left\{\frac{1}{2N_{s}}\sum_{v=1}^{2N_{s}}[F^{2}{\left(v,s\right)}^{\frac{q}{2}}\right\}}^{\frac{1}{q}}$$
  with *q* a parameter that modifies the behavior of *F*_*q*_(*s*). Particularly, the cases of *q* < 0 characterize small fluctuations whereas *q* > 0 refer to larger ones (Kantelhardt et al., 2002). The steps 2–4 are repeated for different time scales *s* in order to construct the log-log plot of *F*_*q*_(*s*) vs. *s*.
- Step 5: Finally, the scaling behavior is described by,
  (4) $$\begin{array}{l} F_{q}\left(s\right)\sim s^{h\left(q\right)}, \end{array}$$
  where *h*(*q*) is an exponent that may depend on *q* and it is called the generalized Hurst exponent. For instance, when *q* = 2, *h*(2) is related to the standard Hurst exponent. For monofractal time series, it is expected that *h*(*q*) remains constant as the value of *q* is changed.

The use of different moments (*q*-values) permits to stablish a relationship between the generalized Hurst exponent *h*(*q*) and the scaling exponent τ(*q*), which is defined via an appropriate partition function in the standard multifractal formalism (Feder, 1988; Kantelhardt et al., 2002). This relationship is given by,

(5) $$\begin{array}{l} \tau \left(q\right)=qh\left(q\right)-1. \end{array}$$

The singularity spectrum *f*(α) can be constructed to characterize the multifractal properties of the time series. Specifically, *f*(α) can obtained from τ(*q*) via the Legendre transform, by taking

(6) $$\begin{array}{l} \alpha =\tau^{\prime }\left(q\right)\text{ and }F\left(\alpha \right)=q\alpha -\tau \left(q\right), \end{array}$$

where α represents the singularity strength of Holder exponent and *F*(α) is the dimension of the subset of the time series that is characterized by α. We also recall that sometimes the multifractal properties are described in terms of the generalized dimensions:

(7) $$\begin{array}{l} D\left(q\right)=\frac{\tau \left(q\right)}{q-1}. \end{array}$$

In order to characterize the multifractal spectrum *f*(α), we resort to the following quantities (Makowiec et al., 2006; Galaska et al., 2008):

- **Width of the spectrum:** distance between the maximum and minimum Holder exponents,
  (8) $$\begin{array}{l} \Delta \alpha =\alpha_{max}-\alpha_{min}. \end{array}$$
- **Left-side width of the spectrum:** distance between α^*^ [which corresponds to $F_{max}\left(\alpha^{*}\right)$] and minimum α value
  (9) $$\begin{array}{l} \Delta \alpha^{left}=\;\alpha_{F_{max}}-\alpha_{min}. \end{array}$$
- **Right-side width of the spectrum:** distance between maximum α and α^*^
  (10) $$\begin{array}{l} \Delta \alpha^{rigth}=\alpha_{max}-\alpha_{F_{max}}. \end{array}$$
- **Global Hurst exponent:**
  (11) $$\begin{array}{l} H_{G}=\frac{1}{2}\left(1+\tau \left(2\right)\right), \end{array}$$
  where τ(2) represents the value of the scaling exponent for *q* = 2.
- **Left-slope and right-slope in** τ**:** linear approximation to the behavior of τ for negative (left) and positive (right) moments *q*.

To further explore the multifractal changes in healthy and FM conditions, we also resort to the magnitude and sign analysis of the RR sequences. Ashkenazy et al. (2001) have reported that scaling properties of the magnitude series are related to the multifractal structure of the origial signal (non-linear properties), whereas scaling sign analysis reveal mostly information about linear correlation of the time series. Briefly, from the increment Δ*RR* series, two new series are constructed: the magnitude series |Δ*RR*| and sign(Δ*RR*), where the function sign(Δ*RR*) is defined to be +1 for Δ*RR* > 0, −1 for Δ*RR* < 0 and 0 for Δ*RR* = 0. The magnitude and sign analysis of heart rate variability have been proved to be useful to deferentiate between pathological changes and certain clinical conditions (Ashkenazy et al., 2001; Schmitt and Ivanov, 2007; Reyes-Lagos et al., 2016).

### 2.2. Patients and data

As previously described (Lerma et al., 2016), we studied 30 women with fibromyalgia. Eligibility criteria for patients were the following: (1) to have fibromyalgia according to the 1990 American College of Rheumatology guidelines; (2) to be free of any medication that could affect autonomic performance including tranquilizers or antidepressants; (3) to be 18–50 years old; (4) to be in the fertile period of their lives with active menstrual cycles, but not to be in their menstrual period the day of the study; (5) to have no comorbid conditions; and (6) to freely agree to participate in the study. Patients were sourced from different rheumatology private practices in Mexico City. For each patient, a control of similar age (±2 years) was recruited. Eligibility criteria for controls were the following: (1) to consider themselves healthy and to have five or fewer fibromyalgia tender points; and (2) Not to be in their menstrual period the day of the study. Controls were medical or paramedical personnel. A rheumatologist examined all prospective participants to ascertain the diagnosis of FM or the healthy status of controls. All participants filled out validated spanish questionnaires for a systematic and comprehensive assessment of their symptoms, including the Fibromyalgia Impact Questionnaire (FIQ) and the Composite Autonomic Symptom Scale (COMPASS). The fibromyalgia group had higher scores of the symptoms than the control group (total FIQ score: 63 ± 16 vs. 10 ± 10, *p* < 0.0001; total COMPASS score: 55 ± 16 vs. 15 ± 11, *p* < 0.0001). A detailed description of all symptoms is described elsewhere (Lerma et al., 2016). FM patients had similar age and body mass index (age = 31 ± 8 years old, body mass index = 23.8 ± 4.4 Kg/m^2^) than healthy participants (31 ± 8 years old, and body mass index (24.4 ± 3.2 Kg/m^2^). All participants signed a written consent form. The study was approved by the Research Committee and by the Bioethics Committee of the Instituto de Cardiología de Mexico.

The RR fluctuations time series of each participant were obtained from an ambulatory 24 h electrocardiogram recording with a Holter monitor (model DMS-307, DMS Inc.). An automated computer program was used to identify the time of occurrence of each heartbeat. Then the difference in time between consecutive heartbeats was calculated (RR interval), and an adaptive filtering method was used to identify and replace the RR intervals that were not originated during normal sinus rhythm (Wessel et al., 2000). The RR fluctuations time series had a mean RR interval of 0.806 ± 0.082 in the FM group and 0.770 ± 0.074 s in the healthy group (*p* = 0.070).

### 2.3. Statistical analysis

Kolmogorov–Smirnov tests were applied to all variables to assess their normal distributions. Variables with normal distribution were compared between groups by Student *t*-tests; variables with no normal distributions were compared by Mann–Withney *U*-tests. Regression analyses of *h*(*q*) vs. *q* were performed to compare values of *h*(*q*) between FM and control groups. For each group, the 95% confidence intervals of each model parameter were estimated in a second order polynomial model [$h\left(q\right)=\beta_{0}+\beta_{1}q+\beta_{2}q^{2}$]. The statistical analysis was performed with SPSS version 21.0.

## 3. Results

**Figure 1:**
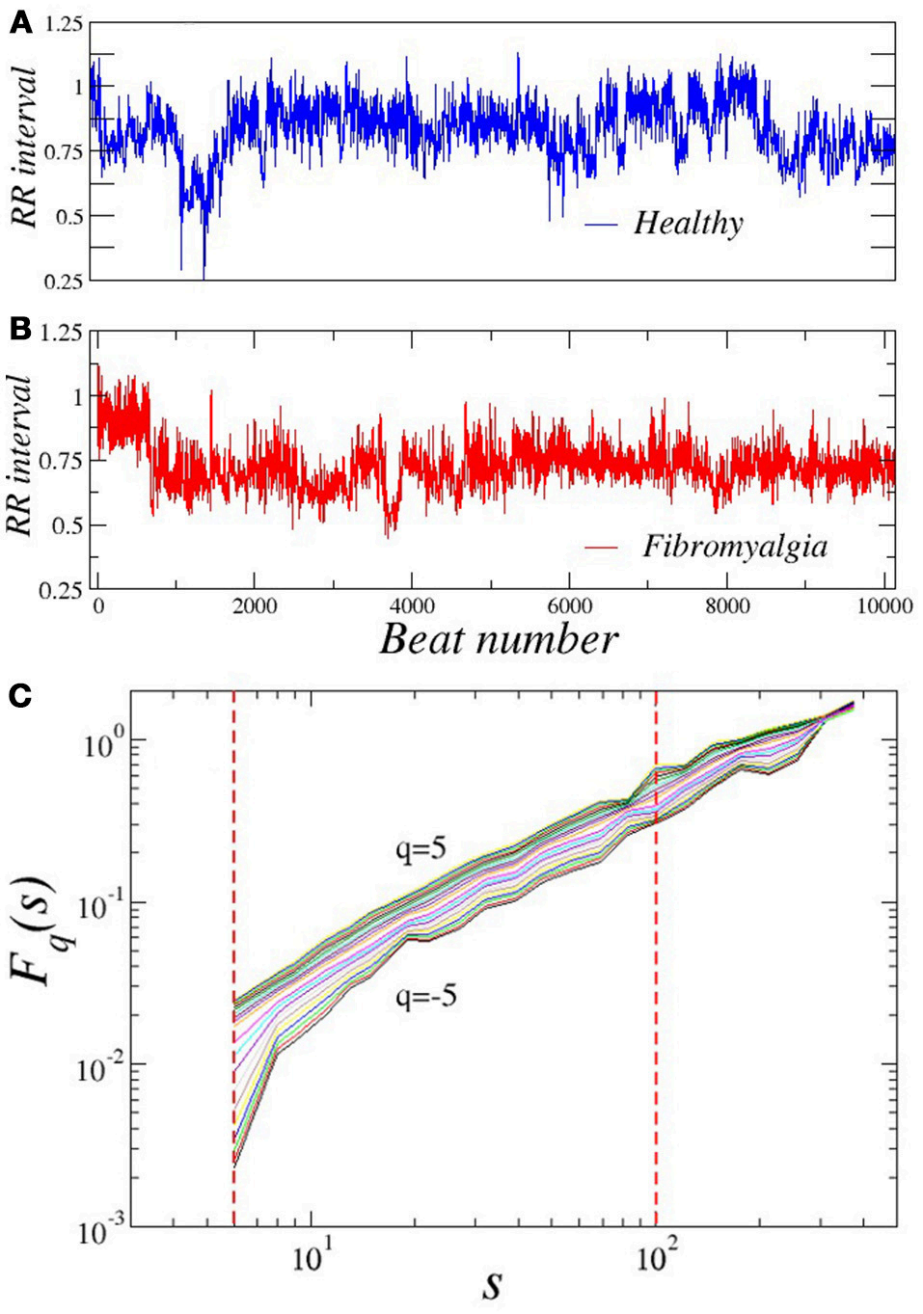
Representative RR time series during timeday of a **(A)** healthy subject and **(B)** patient with FM. Plot of **(C)** *F*_*q*_(*s*) vs. *q* of a RR sequence (healthy subject) for several values of *q*.

**Figure 2:**
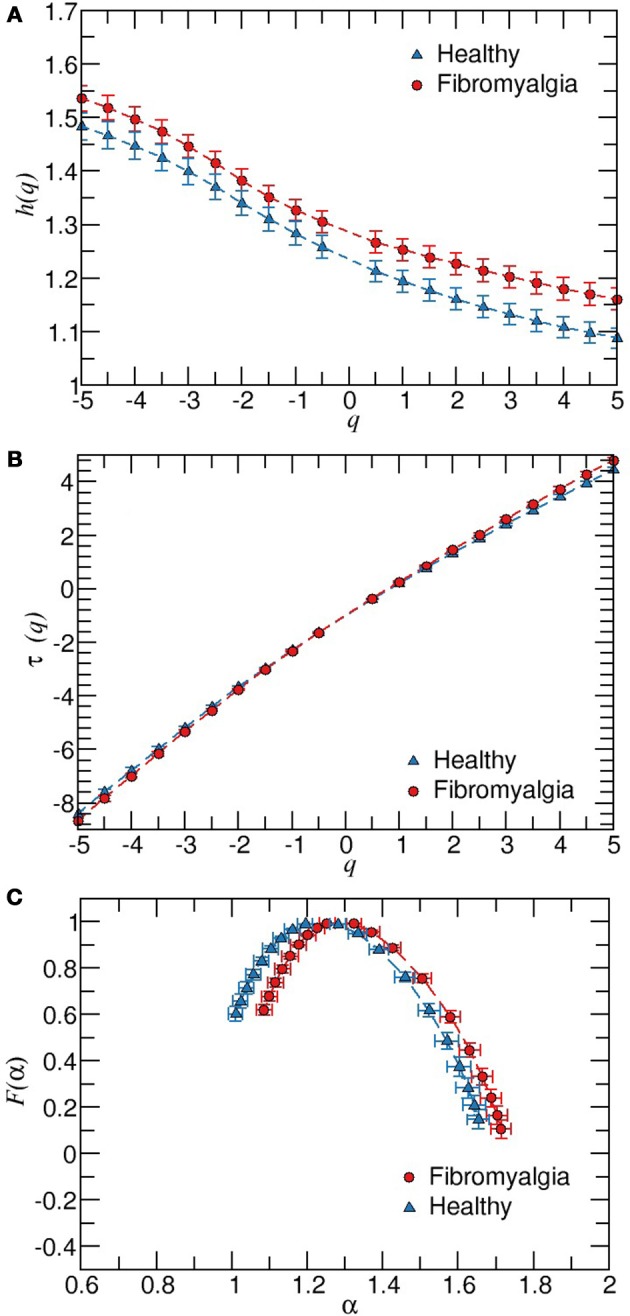
Results of the MFDFA for all healthy and FM subjects. **(A)** Plot of *h*(*q*) vs. *q*. The values of *h*(*q*) are derived from fittings within the interval 6 < *s* < 100 and a time window of 1, 500 RR values during daytime. The estimated regression models were *h*(*q*) = 1.295 − 0.039*q* + 0.003*q*^2^ (for FM patients), and *h*(*q*) = 1.261 − 0.042*q* + 0.002*q*^2^ (for healthy group). In both models, β_0_, β_1_, and β_2_ ≠ 0 with *p* < 0.001 and *R*^2^ > 0.55. The confidence intervals of β_1_ and β_2_ are overlapped in both groups, while there was no overlap in β_0_ of the FM group (1.281, 1.309) with the healthy group (1.226, 1.256). **(B)** Behavior of τ(*q*) for data showed in **(A)**. **(C)** Multifractal spectrum *F*(α) *vs α*. Error bars represent the standard error of the mean.

For both, healthy and FM patients, we performed the multifractal analysis of RR time series from two 5-h segments of the ECG recordings: nighttime (0.00 p.m.–5:00 a.m.) and daytime (2:00 p.m.–7:00 p.m.). These series are illustrated in the Figures 1A,B. First, we explored the behavior of the fluctuactions in the plane *F*_*q*_(*s*) vs. *s* for different values of *q* and for different segment lenghts (from 30 min to 5 h) as shown in the Figure 1C. It is very important to consider the proper range selection of scales *s*, for which the fittings in the fluctuactions *F*_*q*_(*s*) are calculated. In our case, we selected the scale range 6 < *s* < 100 and the length of the segment Δ*t* = 1, 500 ≈ 30 min to apply MFDFA. The values of the local scaling exponents of all subjects are averaged over all segments of the 5 h interval (either at daytime or nighttime). The behavior of *h*(*q*) vs. *q* was determined for *q*-values within the interval [−5, 5], and the multifractal spectrum was then constructed. Figure 2 shows the results of the MFDFA for the healthy and FM groups for daytime records. We observe that the Hurst exponent *h*(*q*) is not independent of *q*, i.e., a similar variation in the average scaling-exponent values as a function of *q* is observed for both groups (the confidence intervals of β_1_ and β_2_ from both groups are overlapped). This is a manifestation of multifractal properties in the time series (Figure 2A). However, the confidence intervals of β_0_ from both groups do not overlap, indicating that the *h*(*q*) values from FM data are consistently higher (i.e., smoother) than the corresponding values of the healthy group. These results are in good concordance with a previous report about monofractal estimation of the scaling exponent [*h*(2)] based on the standard two-point correlation DFA method (Lerma et al., 2016). For healthy subjects, we observe that the behavior of τ(*q*) vs. *q* exhibits slightly more nonlinearity (Figure 2B) than the FM patients, which show more linear τ(*q*) behavior. This indicates that under healthy conditions the dynamics seems to show more multifractal features (Table 1). We find significant differences in Right-slope in τ (*p* < 0.05) between healthy and FM groups, but not significant differences were found for Left-slope in τ (*p* > 0.5). Figure 2C shows the spectra of both groups. We observe that, for healthy subjects, the local Holder exponents cover the range 1 < α < 1.62, while for FM patients the dominant exponents fall within the interval 1.1 < α < 1.7. These results indicate that FM signals are similar to Brownian-type fluctuactions because the interval of the dominant exponents are closer to the 1.5 value.

**Table 1:** Characteristics of the multifractal spectrum in patients with FM and healthy subjects during daytime.

**Parameters**	**Healthy**	**Fibromyalgia**	***p*-value**
Δα	0.6324 ± 0.1809	0.6136 ± 0.1291	0.6565
Δα^*left*^	0.4317 ± 0.1567	0.4320 ± 0.1150	0.9947
Δα^*rigth*^	0.2006 ± 0.1095	0.1816 ± 0.0813	0.4638
*H*_*G*_	1.1595 ± 0.1108	1.2302 ± 0.1089	0.01947
Left-slope in τ	1.5389 ± 0.2122	1.6031 ± 0.2871	0.3459
Right-slope in τ	1.0904 ± 0.1515	1.1770 ± 0.1257	0.0236

*The values of the parameters (mean ± SD) were obtained from fittings within the interval 6 < s < 100 in the logF_q_(s) vs. logs plane. H_G_ stands for global Hurst exponent*.

**Figure 3:**
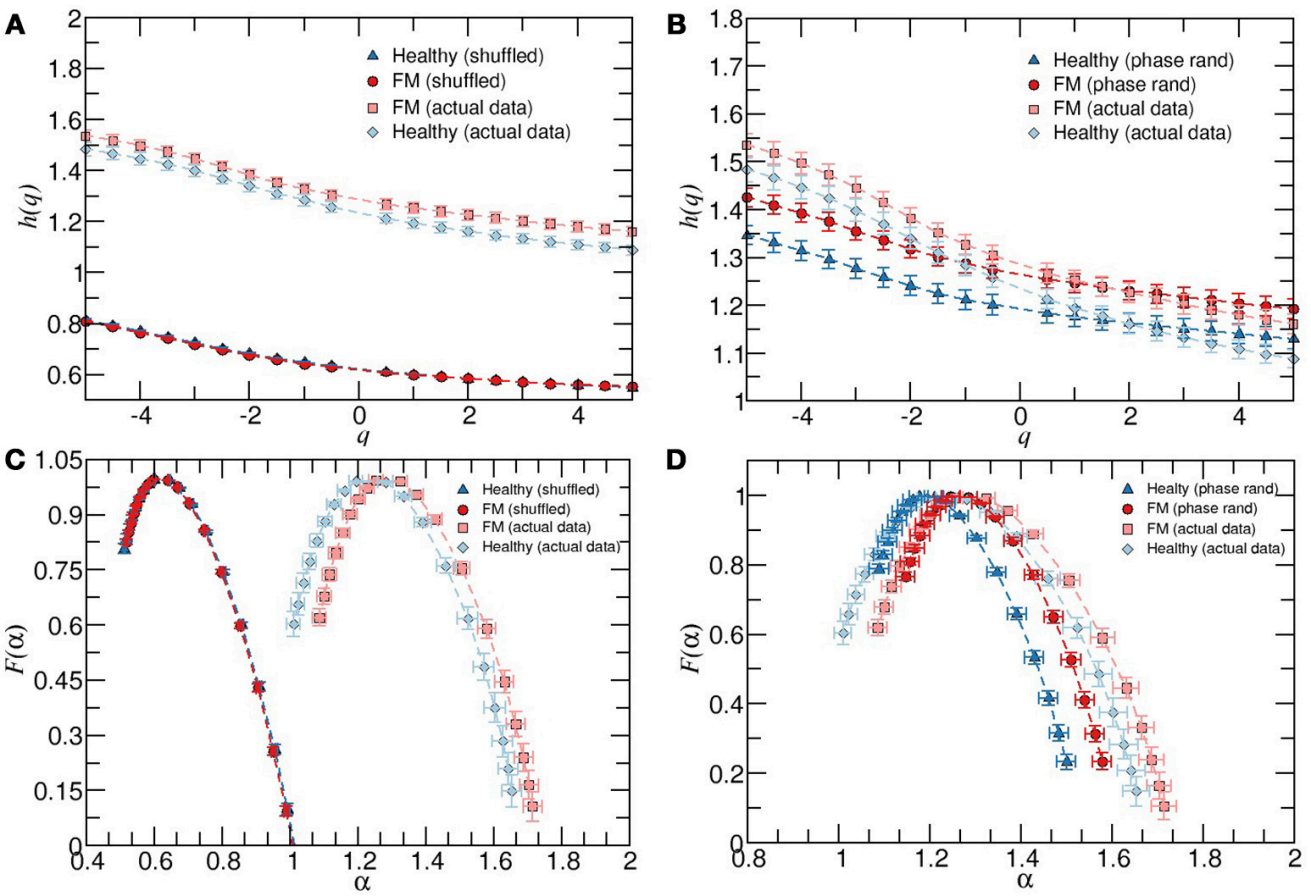
**(A)** Average behavior of *h*(*q*) vs. *q* for healthy subjects, patients with FM and their corresponding shuffled version. **(B)** As in **(A)** but for the phase randomized data. The exponents were calculated for the scaling region 6 < *s* < 100. **(C)** Multifractal spectrum *F*(α) *vs α* for healthy and FM groups and their corresponding shuffled version. **(D)** As in **(C)** but for the phase randomized data, all during timeday. Error bars represent the standard error of the mean.

Next, we also repeated the calculations for nighttime periods. We find that during this period, there are not significant differences between healthy and FM groups regarding the multifractal properties (data not shown). To endorse our findings, we also performed the same analysis on two surrogate data sets derived from each group. First, we shuffled the RR intervals to destroy temporal correlations while preserving the probability distribution. Second, the Fourier transform is applied to the RR intervals sequences, then we randomize the Fourier phases and the inverse Fourier transform is performed to get the surrogate time series. This process preserves the linear properties of the signal (the same power spectrum) but changes the probability distribution of the RR intervals (see Figure 3). We observe that when the temporal two-point correlations are destroyed, the dominant local scaling exponents are close to the 0.5 value for both groups (Figures 3A,C). For phase-randomized data, we found that the width of the multifractal spectrum of the healthy group is narrower than the width corresponding to FM patients (Figures 3B,D), confirming that the contribution of phase correlations to the multifractality is more important in healthy subjects compared to FM patients where the multifractal spectrum suffered a small change, as described above.

**Figure 4:**
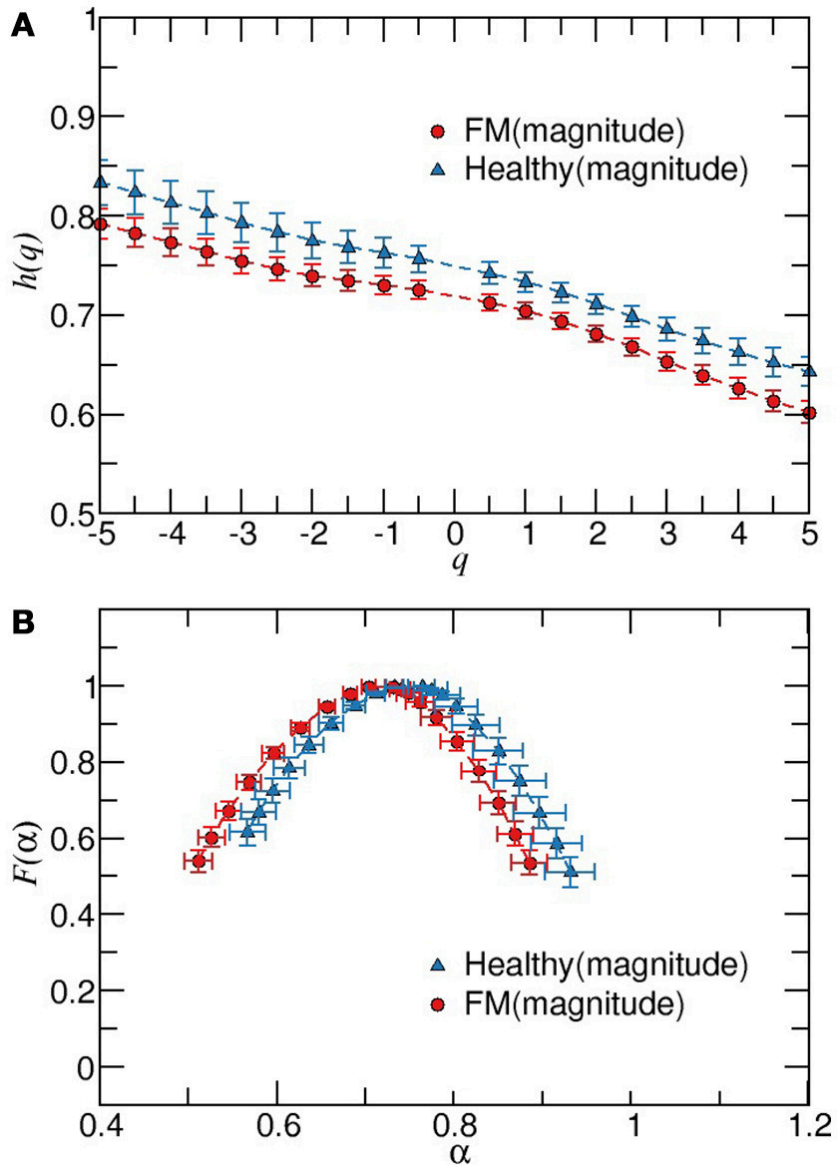
Multifractal detrended fluctuation analysis of magnitude increment sequences of healthy and FM patients during timeday. **(A)** Behavior of *h*(*q*) vs. *q* for scaling exponents obtained from fittings in the scaling region 15 < *s* < 100. *F*(α) vs. α for data showed in **(B)**. Error bars represent the standard error of the mean.

**Figure 5:**
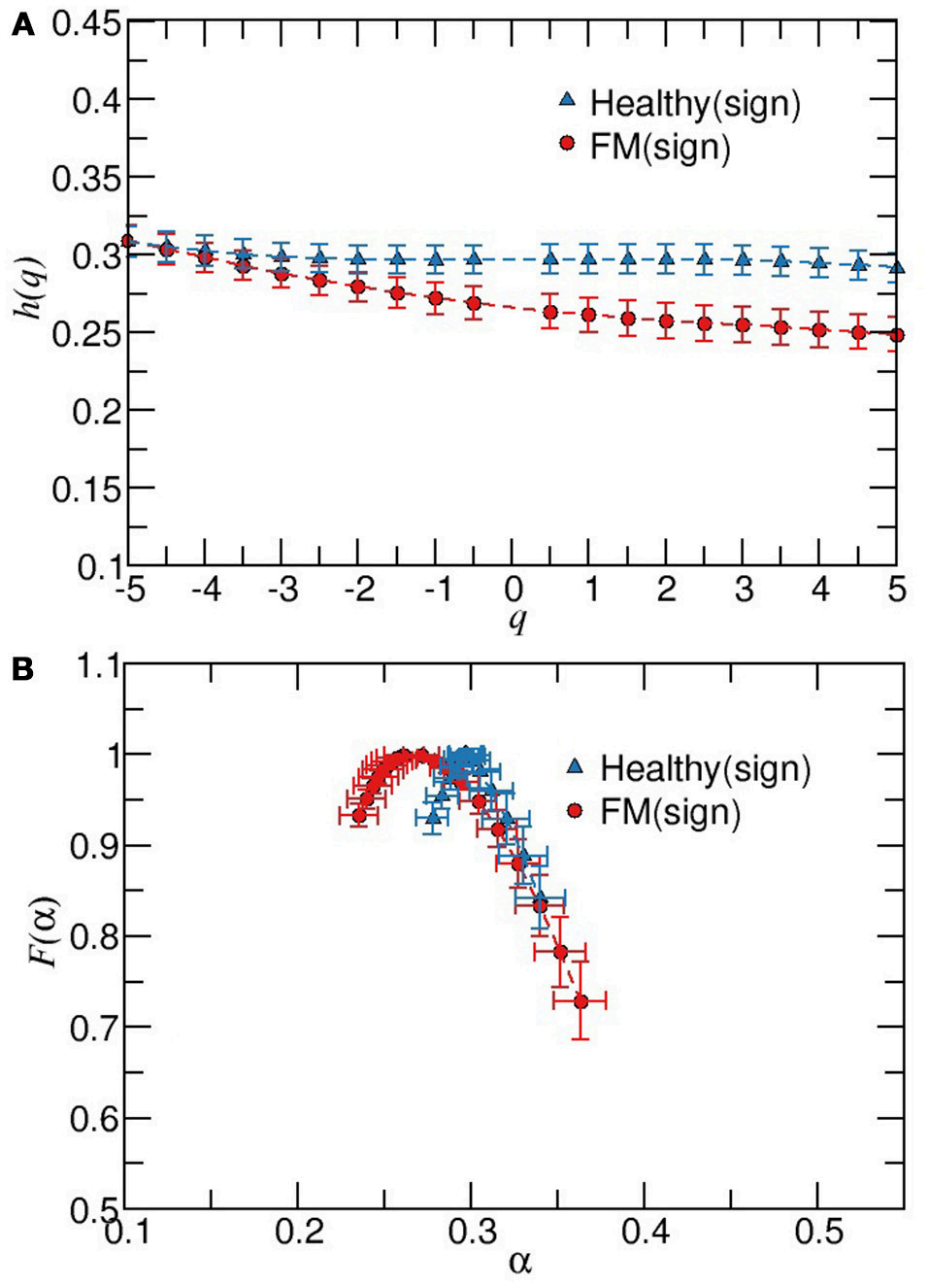
Multifractal detrended fluctuation analysis of sign time series from healthy and patients with FM during timeday.**(A)** Plot of *h*(*q*) vs. *q* for scaling exponents obtained from fittings in the scaling region 15 < *s* < 100. **(B)** *F*(α) vs. α for data showed in **(A)**. Error bars represent the standard error of the mean.

**Figure 6:**
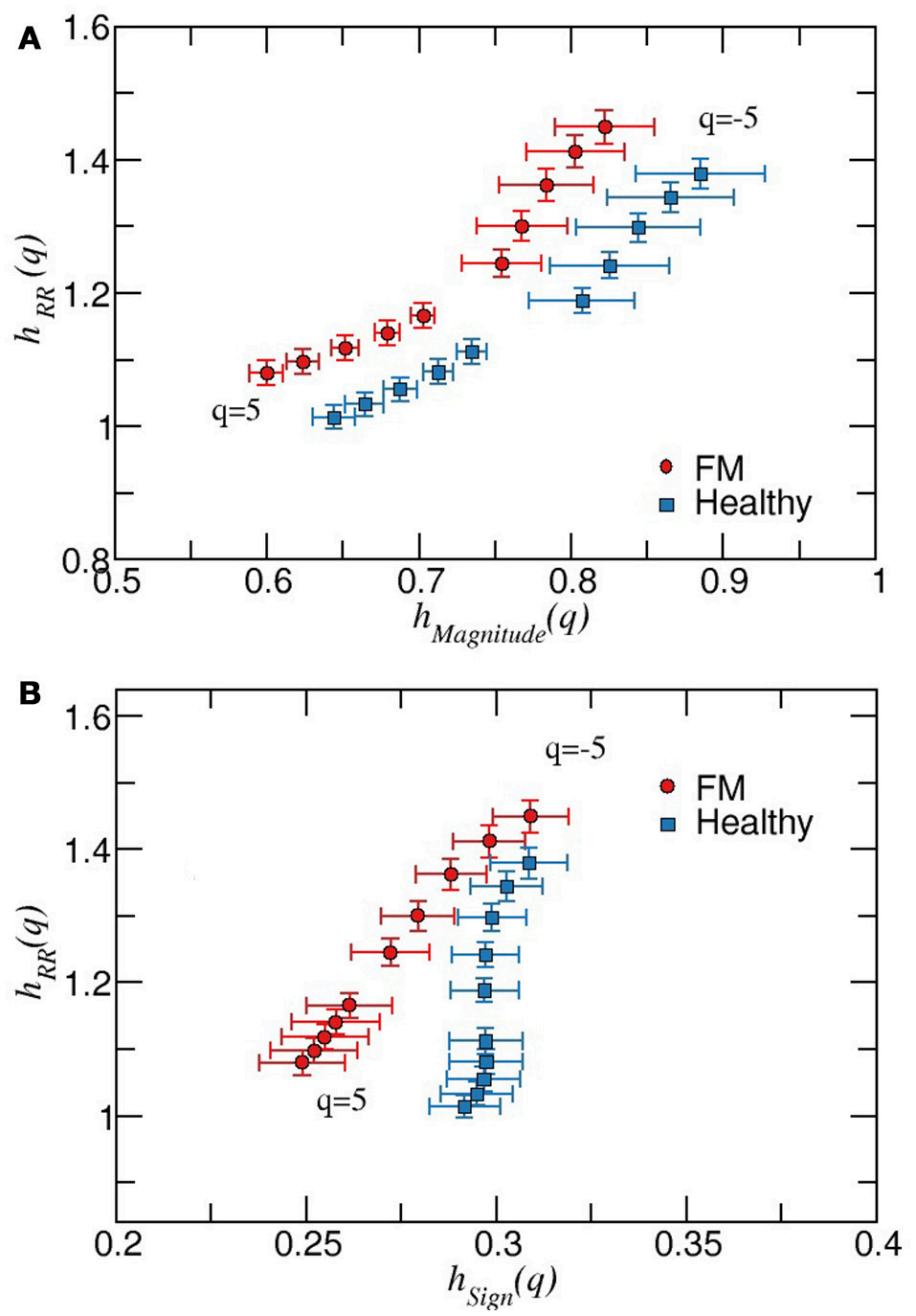
**(A)** Scatter plot of the generalized Hurst exponents *h*_*RR*_(*q*) vs. *h*_*mag*_(*q*), for healthy and FM groups during daytime periods. For both groups, symbols with error bars represent the mean value, and the error bars the corresponding standard error of the mean. We observe that both groups are well separated for the different values of *q*. **(B)** As in **(A)** but for the generalized Hurst exponents *h*_*RR*_(*q*) vs. *h*_*sign*_(*q*), for healthy and FM groups during daytime periods. In this case, both groups are close each other for negative moments while tend to be more separated as *q* increases.

Next, we apply the MDFA to the magnitude and sign series derived from the daytime or nighttime intervals. As it is shown in Figure 4A, the average values of *h*(*q*) of magnitudes series are higher for healthy subjects compared to the FM group. Moreover, the width of the multifractal sprectrum of healthy data is slightly larger than the width of the FM case, confirming that the contribution of nonlinearities is more important under healthy dynamics compared to the FM dynamics (Figure 4B). Figure 5 shows the results of the sign series. We observe that, unlike the previous cases, here FM data lead to a wider multifractal spectrum compared to healthy group. We also remark that the FM group is characterized by a more anticorrelated scaling exponents while the dominant exponents for healthy subjetcs are less anticorrelated. For a better comparison of both groups, the above description of the differences in the multifractal structure between healthy subjects and FM patiens allow us to construct a scatter plot of the generalized Hurst exponent *h*_*RR*_(*q*) of the original RR time series vs. the corresponding Hurst exponent of magnitude *h*_*mag*_(*q*) [or *h*_*RR*_(*q*) vs. sign *h*_*sign*_(*q*)] (Figure 6). We find that, for *h*_*RR*_(*q*) vs. *h*_*mag*_(*q*), two different tendencies are observed for positive and negative moments with a steeper slope in the case of negative moments and both groups are clearly differentiated (Figure 6A). For *h*_*RR*_(*q*) vs. *h*_*sign*_(*q*), sign scaling exponents of healthy data are almost constant for both negative and positive moments while FM patients exhibit variations in both type of scaling exponents and again both groups are separated, specially for positive moments (Figure 6B).

## 4. Discussion

This work presents a thorough exploration of the dynamic behavior of heart rate variability in FM patients from the point of view of multifractality, nonlinearity and directionality. Our main findings are decreased multifractality and non-linearity as well as stronger anticorrelation in FM patients during daytime compared to matched-healthy subjects.

The differences during daytime between groups were mainly identified in two characteristics of the multifractal spectrum (*H*_*G*_ and the right slope in τ) as well as in a larger effect of the phase randomization that provoked a narrower width of the resulting multifractal spectrum for the healthy group. This implied a reduced nonlinearity of heart rate variability in FM patients, which was also confirmed by the multifractal structure of the magnitude series because the generalized *h*_*mag*_(*q*) values of the healthy group were consistently larger for all *q*th values. Moreover, the generalized *h*_*sign*_(*q*) showed consistently smaller values in FM patients, which implies a stronger anticorrelated behavior for all *q*th values (*q* > −4). The multrifractal approach also exhibited that in FM patients, the value of *h*_*sign*_(*q*) changes at different *q*th values, while this variation does not occur in healthy subjects. From a dynamical point of view, whereas a narrower and shifted multifractal spectrum in FM patients reveals smoother Brownian-like fluctuations, in accordance with previous results based on a monofractal analysis (Lerma et al., 2016), their stronger anticorrelated behavior indicates that the fluctuations in heart rate variability tend to alternate the increments and decrements of the RR interval at several scales. Given the well-known effects of periodic trends on the scaling properties (Hu et al., 2004; Schmitt and Ivanov, 2007; Perakakis et al., 2009), such smoother fluctuations in FM patients may arise, among other factors, from the occurrence of periodic breathing patterns that have been observed in FM (Sergi et al., 1999). Future studies are required to asses the potential influence of periodic breathing patterns upon multifractality HRV properties in FM. FM is characterized by a wide range of symptoms and inter-subject variability is usually very high. A strength of our study is the carefully selected patient and control groups. All patients had well-established fibromyalgia, without comorbid conditions and were free of any medication that could affect autonomic nervous system performance. Expert rheumatologists examined all prospective participants to ascertain the diagnosis of FM or the healthy status of controls according to the American College of Rheumatology. Further studies with large samples are needed to assess the generalization of the present findings in more heterogeneous samples of patients

Previous studies have used both time domain and frequency domain heart rate variability analysis. The majority of studies observed lower heart rate variability in FM patients compared to healthy control persons, as well as increased sympathetic activity and a blunted autonomic response to stressors (Meeus et al., 2013). The power spectral analysis of heart rate variability in FM patients have actually shown a sympathetic predominance during all phases of the circadian cycle (Martínez-Lavín et al., 1998), and the time domain analysis indicated a decreased overall variability, which correlated with some FM symptoms (Lerma et al., 2011). In our study, representative multifractality indexes were different between patients and controls during daytime but not at night. A hypothetical explanation for this finding could be that the blunted response to stressors of fibromyalgia patients degrade the multifractal dynamics exhibited by heart rate variability when confronted to the daytime chores. Concerning patients with congestive heart failure, a disease that compromise the cardiovascular dynamics more severely than FM, a monofractal analysis of healthy dynamics vs. patients with congestive heart failure showed scaling exponents of both groups that are closer to each other during sleep phases with a noticeably similar irregular behavior compared to daytime periods (Ivanov et al., 1999b). Far from the reductionist vision of FM as a disease solely caused by chronic sympathetic hyperactivity, we consider that FM and related conditions may result from an overall degraded performance of the autonomic nervous system, which is a complex adaptive system that constitutes the main orchestrator of the stress response system (Martinez-Lavin et al., 2008). From this point of view, previous findings of smoother heart rate variability are consistent with the hypothesis of an altered and more “rigid” autonomic response to stress (Lerma et al., 2016). The current work provides further evidence of this decreased complexity in the autonomic nervous system because the multifractal analysis showed less nonlinearity in FM patients than in healthy subjects. This was observed by a larger *H*_*G*_ and larger right slope in τ (Table 1), which was also confirmed by the surrogate analysis and multifractal analysis of the magnitude series as explained above. Moreover, the multifractal analysis of the sign series showed that FM patients have an increased anticorrelated behavior, which is consistent with the sympathetic predominance of FM according to the stochastic feedback model for random walks (Ivanov et al., 1998). In this model, an increment of anticorrelations is achieved by introducing a dominant attracting factor to set the main heart rate (i.e., instead of concurrent influences from several factors, one modulating factor becomes predominant).

There are other hypotheses beyond the autonomic modulation that have motivated the exploration of changes in the anatomical or functional complexity, which both could be associated with the hyperactivity of the nociceptive system in FM patients. For example, morphometric analysis of different subcortical brain regions in FM patients showed a reduction in the total brainstem volume, which was significantly correlated with the clinical score of tender points (Fallon et al., 2013). From a functional point of view, the interaction between brain areas of FM patients has been assessed through network sensitivity analysis of electroencephalogram data. Two properties of such networks (node degree and frequency) showed significant correlation with chronic intensity pain, suggesting that the central nervous system of these patients has an altered network configuration that may increase hypersensitivity to pain (Lee et al., 2018). However, it remains uncertain if such changes of the central nervous system participate in functional alterations of other regulatory systems, and if this participation modifies effector variables such as the heart rate period.

The multifractal detrended analysis as applied here to magnitude and sign heart rate variability sequences indicated a potential route to evaluate the impairment of the regulatory adaptability because, regardless of characterizing the directionality in small (*q* < 0) or rather large fluctuations (*q* > 0), the level of anticorrelation remained invariant for healthy subjects. Given that our healthy subjects were carefully selected (see section 2), this finding could then imply that such invariance reveals stability because the interplay of different attracting factors (Ivanov et al., 1998) are similar manifested during the course of small or large fluctuations. The current work provides further evidence of this decreased complexity in the autonomic nervous system because the multifractal analysis showed less nonlinearity in FM patients than in healthy subjects. Future studies associating the severity of the disease will determine if this new information has clinical implications and additional analyses remain to be performed to assess this consideration.

## 5. Conclusions

The multifractal analysis of heart rate variability revealed a loss of complexity (i.e., less nonlinearity) and increased anticorrelated dynamical behavior in FM patients, which reinforces the hypothesis of the crucial role of the impaired autonomic nervous systems in the etiology and diverse alterations of FM.

## Author contributions

CR-M participated in the study design, data analysis and interpretation, statistical analysis, and drafting of the article. CL participated in the study design, data collection, data interpretation, and drafting of the article. JE participated in the study design, data interpretation, and drafting of the article. MM-L contributed to the study design, enrollment of patients, and data interpretation. LM-M participated in the study design, enrollment of patients, data collection, and data interpretation. OI contributed to the study design and data interpretation. LG-V contributed to the conception and design of the study, data analysis, data interpretation, and drafting of the article. All the authors did a critical revision and gave their approval to the article.

### Conflict of interest statement

The authors declare that the research was conducted in the absence of any commercial or financial relationships that could be construed as a potential conflict of interest.
